# COVID-19: Academic, Financial, and Mental Health Challenges Faced by International Students in the United States Due to the Pandemic

**DOI:** 10.7759/cureus.41081

**Published:** 2023-06-28

**Authors:** Eniola A Olatunji, Ayobami Ogunsola, Faith Elenwa, Mercy Udeh, Ifeoluwa Oginni, Yeka Nmadu, Timothy Callaghan

**Affiliations:** 1 Health Policy and Management, Texas A&M Health Science Center, College Station, USA; 2 Epidemiology and Biostatistics, Texas A&M Health Science Center, College Station, USA

**Keywords:** academic challenges, financial challenges, patient health questionnaire-4 (phq-4), mental health, united states, international students, covid-19 pandemic

## Abstract

Background and objective

Many international students often face challenges regarding their mental health, finances, and academics. The coronavirus disease 2019 (COVID-19) outbreak may have presented unprecedented challenges to many foreign students in these aspects. Our study examined the academic, financial, and mental health challenges encountered by international students residing in the United States due to the COVID-19 pandemic. It also examined the association between the mental health of the respondents and the academic and financial challenges they encountered.

Method

The study involved international students enrolled at Texas A&M University, who identified themselves as non-US citizens or non-permanent residents. We conducted a cross-sectional study by using Qualtrics® to explore the three domains of the study. Questions included in previous studies were modified to assess the academic and financial status while the Patient Health Questionnaire-4 (PHQ-4) score was used to assess the mental health of the respondents. We presented descriptive statistics for all domains and used an ordered logistic regression to further analyze the effect of the other domains on the mental health of the respondents.

Results

Of the 281 respondents, the majority (79%) experienced challenges with online classes; 91% reported having negative emotions and some students (24%) lost funding due to the pandemic. The inability to pay bills resulted in a three-fold increase in the likelihood of reporting higher mental distress [adjusted odds ratios (aOR): 3.051, 95% CI: 1.665-5.591; p<0.001], and experiencing academic challenges led to a seven-fold increase in the likelihood of reporting higher mental distress (aOR: 7.236, 95% CI: 3.168-12.530; p<0.001).

Conclusion

The COVID-19 pandemic posed a major challenge to international students and its impact on the mental health of the participants was aggravated by concurrent academic and financial hardships.

## Introduction

The coronavirus disease 2019 (COVID-19) pandemic is a global public health emergency that has profoundly affected international students living in the United States. About 1.1 million international students studied in the United States of America during the 2018-2019 school year [1]. Even under normal circumstances, international students are more prone to mental health problems (e.g., depression and anxiety), face a higher risk of financial hardships, and have trouble transitioning to the academic system in the foreign country compared to the citizens and residents studying in that country [2]. Since the COVID-19 outbreak began, unprecedented challenges have threatened the academic, financial, and mental well-being of all students including international students worldwide [3,4]. To better understand the impact of the pandemic on these vulnerable students, this study analyzed the academic, financial, and mental health impact of the COVID-19 pandemic on international students in the United States. To that end, this study relied on an online survey of international students at the College Station campus of Texas A&M University, one of the largest universities in the United States with an international student population of over 5000. For the purpose of this study, the term “international students” refers to students who left their home country to study in the US at the undergraduate or postgraduate level.

International students routinely experience stressors such as language barriers, and financial, cultural, and academic difficulties, as well as challenges in forming new relationships, which may adversely affect their academic performance [5,6]. They may require individualized attention through office hours and tutoring sessions to gain the academic support that they need. However, this opportunity has been severely diminished by the COVID-19 pandemic. A vast majority of domestic and international students were concerned that the pandemic would have a detrimental effect on their academic performance [5,6]. Students reported facing more distractions during online classes, including ineffective engagement with professors and fellow students, extended interruptions from relatives, and increased screen time [7]. Students working on research and class projects have raised concerns about their progress due to social distancing restrictions and the general sense of uncertainty surrounding the outbreak. 

While financial security is a problem for all students, it is even more pronounced for international students [8]. In the US, the employment opportunities for international students are primarily limited to working on campus, and as such, they rely on campus-based employment for supplemental income. When the COVID-19 pandemic forced many universities to move instruction online, many on-campus jobs were cut or reduced in capacity, and several employees were laid off [7]. Since the pandemic began, several students have reported that COVID-19 has affected their internships and part-time employment [7,9]. The economic crisis that ensued significantly affected individuals with low levels of social protection including international students [10,11]. In Australia, for instance, a recent study showed that about half of international students are private renters and depend on paid work to cover their rent, but most of the jobs have been lost since the pandemic began [11]. Consequently, some international students living in private rental accommodations have been unable to pay their rent [11].

In the US, economic stimulus packages were distributed to alleviate some of the financial difficulties brought on by the pandemic; however, international students do not qualify for such financial assistance programs [12]. Research has shown that international students have limited social support systems compared to their domestic peers, mostly because they are living far away from home [13]. Some of these issues result in students facing challenges in terms of adapting culturally to the new country, making new friends, taking care of their financial responsibilities, and finding a balance between work and personal life [13]. They also experience more academic difficulties than their domestic counterparts because of the dissimilarities between the host country's learning styles and those in their home country [13]. During the pandemic, individuals have been required to practice social distancing to reduce the spread of COVID-19, which could give rise to negative emotions such as anxiety and depression among individuals already struggling to adapt culturally and make new friends [14]. With widespread lockdowns around the globe, international students could further experience more unmet psychological needs due to this lack of social support and necessary isolation [15].

With most colleges transitioning to online instruction, students staying at home might have to adopt a different sleeping pattern leading to reduced sleep quality, thereby elevating stress levels and anxiety about their academic and employment prospects [14]. A study conducted in China showed that the prevalence of anxiety and depression increased among students during the first phase of the COVID-19 pandemic [14]. Furthermore, there has been a surge in incidents of microaggression and even blatant discrimination toward international students, especially those of Asian origin, due to the perception that they might be potential COVID-19 carriers [16]. Consequently, such students are at an increased risk of depression, anxiety, and other mental health challenges.

Given these various challenges faced by international students, it is important to identify them and develop actionable insights to improve the well-being of international students during and after the coronavirus pandemic. In this study, we aim to examine the academic, financial, and mental health challenges experienced by international students at a large academic institution in the United States. Furthermore, we aimed to assess the relationship between the mental health challenges experienced by students and the academic and financial challenges during the pandemic.

## Materials and methods

Study design

A convenience sampling technique was used to administer an online cross-sectional survey by using Qualtrics® to assess the mental health, financial, and academic status of international students at a large US university. The survey was made available in the English language and was disseminated using the Texas A&M University bulk email system, which allows for the targeting of international students. In this study, we recruited individuals enrolled at the undergraduate or graduate levels who were non-US citizens or non-permanent residents. Recruitment emails contained links to the online survey and were sent out five times between November 16, 2020, and January 14, 2021, to maximize response rates. Prior to the surveys being administered, a pilot version was pretested among 20 international students from the same university to determine the degree to which the survey questions were comprehensible and to enhance the validity of the study. The pretested survey questionnaire was improved based on the feedback provided by the pretest subjects, but the pretest result was not included in the final analysis.

Study participants

The inclusion criteria were as follows: students currently enrolled at Texas A&M University at undergraduate, master's, or Ph.D. levels and registered as international students. US citizens or permanent residents in the US were excluded from the study. After the recruitment emails were sent, 281 participants who met the study criteria responded to the survey. Study participation was voluntary and before participants began the survey, they were provided an electronic informed consent form, information about the nature and purpose of the study, and information about the ethical approval obtained from the Texas A&M Institutional Review Board.

Study variables and measures

The study consisted of 42 questions and was required to be completed in approximately 10 minutes. The questions can be categorized into the following sections: (1) 10 questions about participants’ demographics, (2) Eight questions assessing the academic challenges associated with the COVID-19 pandemic, (3) 12 questions assessing the financial challenges posed by the COVID-19 pandemic, and (4) 12 questions assessing the mental health challenges linked to the COVID-19 pandemic. To assess the effect of the pandemic on respondents, questions assessing mental health were framed using the Patient Health Questionnaire-4 (PHQ-4), while questions on academic and financial challenges were adapted from previous studies [17-19].

Specifically, the mental health challenges were explored by employing PHQ-4-types questions such as the following: "Since March, how often have you felt depressed due to the pandemic?" and non-PHQ-4-type questions such as the following: "I feel the pandemic has affected my mental health". These had to be answered with the categorical options of "none of the time", "some of the time", "most of the time", and "all of the time" and graded on the Likert scale from "strongly agree" to "extremely disagree", respectively. In addition, a composite PHQ-4 score was created by combining the responses to each PHQ-4 component question, which was subsequently used as a dependent variable in an ordered logistic regression. The PHQ-4 has been tested and validated in previous studies. All four questions in the PHQ-4 were used. The PHQ-4 has been previously tested and validated in similar populations and other populations where it has been shown to have a 0.8 association with mental health [20]. PHQ-4 operating characteristics were estimated and area under the curve (AUC) values were 0.835 and 0.787 for anxiety and depression, respectively [20]. To explore the academic impact of the pandemic, an example of the questions included was as follows: “I feel the COVID-19 pandemic affected my academic performance”. This had to be answered based on the Likert scale with the options ranging from "strongly agree" to "extremely disagree". Another question, "Have you adapted to the new methods of teaching and learning?" with response options of "agree, disagree, partially, and undecided" was used to further understand the academic impact. The assessment of the financial impact of the pandemic was performed by asking questions such as the following: "Do you have upcoming bills that you are unable to pay due to the COVID-19 pandemic?" This had to be answered categorically as "yes", "no", or "prefer not to say". Another question asked, "In what way has COVID-19 affected your finances?" Responses to this question included "reduced income", "increased income", "not affected", and "prefer not to say".

Data analysis

Descriptive statistics (sample size=281) were used to report the distribution of the demographic factors and all other survey component responses. Furthermore, an ordered logistic regression analysis was conducted using the PHQ-4 scores as a categorical dependent variable in four levels. The total score ranged from 0 to 12, with the ordered categories identified as "none" (0-2), "mild mental distress" (3-5), "moderate mental distress" (6-8), and "severe mental distress" (9 -12) [17]. The sample size for the ordered logistic regression was 243, representing the complete case analysis. The difference in sample size is due to the exclusion of respondents who did not provide complete answers to the PHQ-4 questions used as the dependent variable in the model. The proportional odds assumption was not violated (score test p=0.331) and there was no multicollinearity among the covariates as none had a variance inflation factor (VIF) of more than 2. We hypothesized that higher challenges with academic performance and finances would be associated with a higher score on the PHQ-4 scale among the respondents. For the model selection, both backward and forward model selections were performed and resulted in the same final variable selection. The initial model included 13 variables and the final model comprised four variables. The data analysis was done using the SAS 9.4 software [21].

Furthermore, since all the questions in a questionnaire/survey do not always directly translate into variables to be included in a model, the reasonableness of the questions to be included in the model was assessed besides including it as a question in the questionnaire. To develop a parsimonious model, we initially ran a univariate analysis for the 42 variables (39 after combining the PHQ-4 variables) and selected the variables that were either statistically significant with p ≤0.05 or were deemed necessary to answer our research question. The final 13 variables included to create a parsimonious model were as follows: PHQ-4 score; current marital status; gender; race; ethnicity; the degree the student is working toward; current employment status; the current status of funding provided by Texas AM; annual family income; the way in which COVID-19 has affected the student's finances; whether the student is unable to pay past/upcoming bills due to COVID-19; whether COVID-19 has negatively affected the student's academic performance; and whether the student has lost a specific funding source due to COVID-19.

## Results

A total of 281 international students (i.e., non-US citizens or non-permanent residents) were included in the analysis; 51% were female, with Asians representing the largest proportion of international students in the survey (56%) (Table 1). Regarding the type of degrees, the largest percentage of students (47%) were working towards their doctorate and 20% of participants were undergraduates. The median age was 26 years [interquartile range (IQR): 23-30 years]. Most of the respondents were single (76%), 21% were married, 0.1% were divorced, and 3% were in a domestic partnership (legal relationship, and domestic cohabitation without marriage). Of note, 59% of the participants were employed, with graduate assistantship constituting the most common employment type (Table 1).

**Table 1:** Demographic characteristics IQR: interquartile range; TAMU: Texas A&M University; GTA: graduate teaching assistant; RA: research assistant; GA: graduate assistant

Variables	Categories	Values (n=281)
Sex, n (%)	Male	137 (49)
	Female	143 (51)
	Others	1 (0.4)
Age, median (IQR)		26 (23–30)
Race, n (%)	Black	24 (9)
	Asian	157 (56)
	White	57 (20)
	Others	42 (15)
Marital status, n (%)	Single	207 (76)
	Married	57 (21)
	Living with a partner	9 (3)
	Divorced	1 (0.4)
Ethnicity, n (%)	Hispanic/Latino/Spanish origin	49 (18)
	Not Hispanic/Latino/Spanish origin	230 (82)
Current educational level, n (%)	Undergraduate degree	54 (20)
	Masters degree	90 (33)
	Doctoral degree	128 (47)
Current employment status, n (%)	Employed (20 hours/week)	161 (59)
	Looking for work	5 (2)
	Not employed and not looking for a job	56 (21)
	Unable to work	37 (14)
	Retired	13 (4)
Are you currently being funded by the university? n (%)	Funded, GA (teaching or research)	119 (44)
	Funded, TAMU scholarship	34 (13)
	External funding only	21 (8)
	Not funded	79 (29)
	GTA/RA and external funding	1 (0.4)
	GTA/RA and TAMU scholarship	12 (4)
	TAMU scholarship and external funding	4 (1)
	GA/TAMU scholarship/external funding	1 (0.4)
4-level annual family income categories, n (%)	$0–$10,000	35 (13)
	$10,001–$25,000	103 (38)
	$25,001–$40,000	51 (19)
	$40,000 and above	77 (29)
Do you have a support group? n (%)	Yes	136 (55)
	No	113 (45)

Table 2 explores the academic challenges faced by international students due to the COVID-19 pandemic. A large proportion of the respondents (62%) expressed that they were not comfortable participating in a face-to-face mode of teaching (Table 2). Meanwhile, a considerable proportion of the respondents (45%) were somewhat satisfied with the university's response to the pandemic. The main challenge with regard to adapting to the hybrid mode of teaching provided by the university was fear of contracting the COVID-19 virus (32%) and only 21% reported facing no challenge regarding the hybrid model. Other challenges identified included internet access (11%), class schedules (15%), and challenges with using online meeting platforms (19%). Overall, 73% of the students agreed that the pandemic had negatively affected their academic performance, while 13% were neutral and 14% disagreed. The majority of the respondents (84%) reported that the pandemic has disrupted their ability to study with their peers; 73% of the survey participants reported that COVID-19 had a negative impact on their academic performance, and 21% faced no challenges in using hybrid learning. In comparison, 79% of the students reported that internet access, class schedule, and utilizing online classroom meeting platforms were a challenge. Over 50% of respondents were satisfied with the academic support they received from their professors during the pandemic; however, a quarter of the respondents were not satisfied with the academic support from the university administration during the pandemic.

**Table 2:** Academic challenges faced by the respondents due to the COVID-19 pandemic COVID-19: coronavirus disease 2019; TAMU: Texas A&M University

Variable	Categories	N (%), n=281
How satisfied are you with the University's response to the pandemic?	Extremely satisfied	35 (14)
	Somewhat satisfied	112 (45)
	Neither	41 (17)
	Somewhat dissatisfied	39 (16)
	Extremely dissatisfied	21 (8)
Do you feel comfortable participating in face-to-face classes this semester?	Yes	96 (38)
	No	157 (62)
Are you comfortable with the hybrid option for classes?	Extremely comfortable	77 (30)
	Somewhat comfortable	98 (39)
	Neither	39 (15)
	Somewhat uncomfortable	28 (11)
	Extremely uncomfortable	11 (4)
What has been your biggest challenge in moving to a hybrid setting?	Internet access	29 (11)
	Class schedule	37 (15)
	Fear of contracting COVID-19	86 (34)
	Using the online meeting platform	47 (19)
	No challenge	52 (21)
I feel that COVID-19 has negatively affected my academic performance	Strongly agree	89 (35)
	Somewhat agree	96 (38)
	Neither agree nor disagree	33 (13)
	Somewhat disagree	18 (7)
	Strongly disagree	16 (6)
I feel that COVID-19 has disrupted my ability to study with my peers	Strongly agree	141 (56)
	Somewhat agree	68 (27)
	Neither agree nor disagree	18 (7)
	Somewhat disagree	13 (5)
	Strongly disagree	11 (4)
I am satisfied with the academic support from my professors during the pandemic	Strongly agree	70 (28)
	Somewhat agree	97 (38)
	Neither agree nor disagree	39 (15)
	Somewhat disagree	31 (12)
	Strongly disagree	16 (6)
I am satisfied with the academic support from the TAMU administration during the pandemic	Strongly agree	46 (18)
	Somewhat agree	88 (35)
	Neither agree nor disagree	63 (25)
	Somewhat disagree	29 (12)
	Strongly disagree	25 (10)

While understanding the academic impact of the pandemic is important, it is equally important to understand the financial impact of the pandemic on international students, as both can have a profound effect on their mental health. As shown in Table 3, which focuses on student finances, we found that about 52% of the respondents had their income reduced due to the pandemic with the loss of part-time jobs being the most common cause (Table 3), while 46% reported no effect on their income. About 24% of the respondent reported losing funding sources due to the COVID-19 outbreak. Although a greater proportion of respondents did not have unpaid bills due to the pandemic, 23% of respondents were unable to pay past/upcoming bills. The most commonly identified financial issues were related to rent and tuition. Only 24% considered the COVID-19 pandemic a substantial financial threat to their household income, while 46% considered the outbreak a moderate threat; 24% considered it not much of a threat and 6% considered the outbreak no threat to their household finances.

**Table 3:** Financial challenges faced by the respondents due to the COVID-19 pandemic COVID-19: coronavirus disease 2019

Variable	Categories	N (%), n=281
In what way has COVID-19 affected your finances?	Increased income	5 (2)
	Reduced income	138 (52)
	Not affected	124 (46)
How much of a threat would you say the coronavirus outbreak is to your household finances?	Substantial threat	65 (24)
	Moderate threat	121 (46)
	Not much of a threat	63 (24)
	Not at threat at all	15 (6)
Are you unable to pay past/upcoming bills due to COVID-19?	Yes	61 (23)
	No	203 (77)
Unable to pay grocery bills	No	264 (94)
	Yes	17 (6)
Unable to pay rent	No	247 (88)
	Yes	34 (12)
Unable to pay tuition fee	No	249 (89)
	Yes	32 (11)
Unable to pay student loans	No	257 (91)
	Yes	24 (9)
Unable to pay health insurance	No	263 (94)
	Yes	18 (6)
Have you lost a specific funding source due to COVID-19?	Yes	64 (24)
	No	198 (76)
Loss of research funding due to the pandemic	No	264 (94)
	Yes	17 (6)
Loss of part-time job due to the pandemic	No	242 (86)
	Yes	39 (14)
Loss of grants due to the pandemic	No	272 (97)
	Yes	9 (3)

The COVID-19 pandemic led to mental health challenges among international students, as shown in Table 4. The table summarizes these challenges based on the responses to the PHQ-4 questionnaire. This section also included responses regarding concerns about immigration status and future job prospects of the respondents in the US due to the pandemic. In our study, we observed significant concerns among the participants regarding their immigration status, with 71% of respondents reporting that the pandemic is likely to affect their immigration visa and 80% reporting that the pandemic will negatively affect their future job prospects in the United States. About three-quarters of the respondents (71%) stated that the pandemic had worsened their mental health, and 65% reported that their relationship with their American friends have been disrupted. There were also reported disruptions in sleep patterns (69%) and eating patterns (65%). About a quarter of the respondents felt anxious nearly every day since March 2020. Only 14% of the respondents reported being discriminated against because of their nationality; 27% were neutral while 59% reported no experience of discrimination based on their nationality during this period. More than a third (37%) of the respondents agreed that they felt safe walking around campus while almost a similar proportion (40%) disagreed. The remaining 22% neither disagreed nor agreed that they felt safe walking around campus.

**Table 4:** Mental health challenges faced by the respondents due to the COVID-19 pandemic COVID-19: coronavirus disease 2019

Variable	Categories	N (%), n=281
Since March, how often have you felt nervous, anxious, or on the edge?	Not at all	22 (9)
	Several days	123 (49)
	More than half the days	44 (18)
	Nearly everyday	60 (24)
Since March, how often have you not been able to stop or control worrying?	Not at all	46 (19)
	Several days	104 (42)
	More than half the days	52 (20)
	Nearly everyday	46 (19)
Since March, how often have you had little interest or pleasure in doing things?	Not at all	37 (15)
	Several days	122 (49)
	More than half the days	55 (22)
	Nearly everyday	35 (14)
Since March, how often have you been feeling down, depressed, or hopeless?	Not at all	45 (18)
	Several days	112 (45)
	More than half the days	50 (20)
	Nearly everyday	41 (17)
I feel overwhelmed by the health guidelines put in place	Strongly agree	63 (25)
	Somewhat agree	78 (31)
	Neither agree nor disagree	55 (22)
	Somewhat disagree	36 (14)
	Strongly disagree	19 (8)
I feel the pandemic has disrupted my sleeping pattern	Strongly agree	114 (46)
	Somewhat agree	57 (23)
	Neither agree nor disagree	33 (13)
	Somewhat disagree	20 (8)
	Strongly disagree	23 (9)
I feel the pandemic has disrupted my eating pattern	Strongly agree	104 (42)
	Somewhat agree	61 (25)
	Neither agree nor disagree	33 (13)
	Somewhat disagree	29 (12)
	Strongly disagree	20 (8)
I feel the pandemic has affected my relationship with my American friends	Strongly agree	87 (35)
	Somewhat agree	75 (30)
	Neither agree nor disagree	57 (23)
	Somewhat disagree	13 (5)
	Strongly disagree	15 (6)
I feel safe walking around campus during the pandemic	Strongly agree	26 (10)
	Somewhat agree	67 (27)
	Neither agree nor disagree	55 (22)
	Somewhat disagree	60 (24)
	Strongly disagree	40 (16)
I have been discriminated against because of my nationality during the pandemic	Strongly agree	13 (5)
	Somewhat agree	23 (9)
	Neither agree nor disagree	67 (27)
	Somewhat disagree	29 (12)
	Strongly disagree	115 (47)
I feel the pandemic is likely to affect my immigration visa	Strongly agree	100 (40)
	Somewhat agree	76 (31)
	Neither agree nor disagree	41 (17)
	Somewhat disagree	12 (5)
	Strongly disagree	18 (7)
I feel the pandemic will reduce my future job prospects in the US	Strongly agree	121 (49)
	Somewhat agree	77 (31)
	Neither agree nor disagree	28 (11)
	Somewhat disagree	11 (4)
	Strongly disagree	10 (4)

Table 5 illustrates the model outcome using the PHQ-4 as an ordinal dependent variable where a score of 0-2 represents the absence of mental distress, 3-5 represents mild mental distress, 6-8 represents moderate mental distress, and 9-12 represents severe mental distress. The proportional odds assumption was not violated, and hence the estimate of the slope between each pair of outcomes across two response levels is assumed to be the same irrespective of the cut-off level. The ordered logistic regression using the backward model selection method showed differential PHQ-4 results by race. Black [adjusted odds ratios (aOR): 0.091, p<0.001] and Asian (aOR: 0.390, p=0.012) international students had significantly lower odds of reporting a higher PHQ-4 score category compared to White international students (Table 5). Furthermore, people who could not afford to pay past or upcoming bills had a statistically significant 3.01 higher odds of reporting a higher PHQ-4 score category compared to those who did not report any issues with paying bills. Respondents who reported that the pandemic affected their academic performance had higher odds of reporting a higher PHQ-4 score by seven times compared to those who were neutral. Meanwhile, those who strongly disagreed with the pandemic having any effect on their academic performance had lower odds of reporting a higher PHQ-4 score (aOR: 0.228, p=0.025).

**Table 5:** Crude and adjusted odds ratio of the ordered logistic regression model using the Personal Health Questionnaire 4 (PHQ-4) score as the dependent variable *Statistically significant

Variables (n=243)	Crude odds ratio (95% CI)	P-value	Adjusted odds ratio (95% CI)	P-value
Race				
White	Reference		Reference	
Asian	1.656 (0.919, 2.983)	0.1299	0.390 (0.188, 0.811)	0.012*
Black	9.699 (3.451, 17.263)	<0.001*	0.091 (0.027, 0.304)	<0.001*
Others	1.476 (0.679, 3.206)	0.1276	0.553 (0.239, 1.279)	0.166
Ethnicity				
Non-Hispanic	Reference		Reference	
Hispanic	0.924 (0.511, 1.672)	0.7950	2.018 (0.882, 4.618)	0.0964
Bills (could not afford to pay past or upcoming bills)				
No	Reference		Reference	
Yes	0.375 (0.215, 0.653)	<0.001*	3.051 (1.665, 5.591)	<0.001*
Academic performance				
Neutral	Reference		Reference	
Strongly agree	0.118 (0.052, 0.265)	<0.001*	7.236 (3.168, 12.530)	<0.001*
Somewhat agree	0.455 (0.209, 0.990)	0.0025	1.796 (0.816, 3.900)	0.146
Somewhat disagree	2.607 (0.847, 8.026)	0.009*	0.339 (0.107, 1.074)	0.066
Strongly disagree	4.734 (1.367, 12.399)	<0.001*	0.228 (0.068, 0.835)	0.025*

## Discussion

The COVID-19 pandemic has significantly transformed the educational prospects and opportunities for many students globally, particularly international students [3]. The many unexpected changes, in addition to the long-term stress of the pandemic, have posed various levels of financial, academic, and mental health challenges [2]. In this study, we examined the impact of the COVID-19 pandemic on international students’ financial, academic, and mental well-being at Texas A&M University. The findings from this study suggest that most international students have experienced financial, academic, and psychological challenges due to the COVID-19 pandemic.

The e-learning method implemented to continue with learning activities during the pandemic in many parts of the world has several limitations, such as social isolation, reduced physical student-teacher contact, and issues related to internet connectivity [22]. A significant concern among international students pertained to retaining their international student status by taking the required number of face-to-face classes instead of online courses. This sudden interruption in educational activities might have led to pressure and fear of losing legal temporary immigration status among these students, thus negatively impacting their academics. The closure of embassies and the United States Citizenship and Immigration Services (USCIS) could have also put international students under more stress, especially those who needed passport renewals and Optional Practical Training (OPT) approvals [23]. Additionally, OPT and Curriculum Practical Training (CPT) opportunities became more challenging to obtain or got canceled. In line with our study, another study found that most participants expressed concerns regarding academic performance [7]. However, almost half of the participants reported lower stress levels related to academic pressure and class workload since the beginning of the pandemic [7]. Actions taken by professors, such as reduced course loads and open-book examinations, could also have contributed to alleviating the stress.

Our study found differences among races/ethnicities in terms of mental health outcomes. International students belonging to minority groups (Blacks and Asians) have reported fewer mental health challenges as TAMU has one of the biggest international student communities in the US. Also, these two groups come from a more communal society where limited resources are shared, and the focus is on the collective rather than the individual, in contrast to White students, who may come from a relatively more individualistic society with an emphasis on the individual over the group and with less sharing of resources. Hence, their societal norms carried over to the US in a location with a sizable international student representation may have further enhanced the propensity for ethnic connectedness [24]. This could have helped minority international students to cope better with the mental impact of the pandemic.

COVID-19 also had adverse impacts on the economy of the United States as well as on individuals. A previous study reported a similar economic impact due to national health crises during epidemics and reported that loss of income sources could lead to anxiety about paying rent and tuition fees [25]. This financial challenge among international students could be attributed to living far away from their loved ones, not having a robust support network, not finding a place to live when dormitories were closed, and losing jobs. In some countries, banking services could not be accessed due to shutdowns, and hence family members were not able able to send money. The United States Government took many measures to help the populace of the country, which included stimulus packages to offer financial relief to American students and citizens. Only US tax residents who met certain conditions were eligible to receive the payment. While tax residency is separate from US citizenship, most international students are classified as nonresidents for tax purposes except if they satisfy the Substantial Presence Test [26]. Our survey showed that many students were unable to buy groceries and pay rent at some point during the pandemic. Therefore, efficient and robust social and financial support is necessary for international students during crises like public health emergencies. While most (59%) of the participants were satisfied with the university’s response to the pandemic, there is still room for improvement for authorities to implement more robust policies to reduce the impact of such crises by providing timely and consistent interventions and relief to international students. Universities are responsible for improving their support to vulnerable groups in particular, and populations at a higher risk of negative impact from crises [27].

Of note, only 9% of international students reported feeling no form of anxiety and 19% reported no uncontrollable worry in this study. However, another study conducted to assess the psychological impact of COVID-19 among university students (in general and across seven states) in the United States found that a much smaller proportion (1%) of students reported positive changes due to the COVID-19 pandemic [28]. Some COVID-19-related stressors, such as economic stressors and academic interruptions, were positively associated with anxiety symptoms during the epidemic. Another study found that separation from classmates during the COVID-19 epidemic made students stressed and anxious and previous evidence suggests that college students usually develop attachment to their social group [29]. Reports from China revealed that institution closure, social distancing, travel warnings, and bans disrupted routine life and resulted in anxiety [29].

Overall, it appears that academic and financial challenges affected students’ mental states. It is more likely for a student with academic and economic challenges to be anxious and depressed, emphasizing the association between mental health status and academic/financial status. We recommend that universities boost their outreach to vulnerable populations at risk of high levels of negative impact from COVID-19 [30]. Furthermore, universities can devise more personalized learning methods and international student services and enhance virtual social connections with colleagues and the university community. These efforts may help at-risk groups succeed academically, foster stronger relationships, and improve their sense of belonging during remote learning [31].

There are several limitations to this study. Firstly, this study was based on a convenience sample and may not be representative of the entire international student population at Texas A&M University. Out of over 5,000 international students on campus, only 281 responded to the survey, as participation was voluntary. For the ordered logistic regression, 243 respondents completed the response with regard to the specified dependent variable - PHQ-4. Since certain sensitive survey questions may not always receive a full response, and as per the ethical considerations relating to survey administration, respondents were provided the option not to respond to questions they may not wish to answer. Hence, for the descriptive analysis table, we used a sample size of 281 to preserve vital and valid information provided by the respondents, while a complete cases analysis (243) was used for the ordered logistic regression. As this was our dependent variable, it was important that the respondents gave full responses to the four component variables that were later combined to form the PHQ-4 score variable used in the analysis. Furthermore, adhering to the algorithm by Detorri et al., a complete case analysis as conducted was deemed best for our analysis due to the proportion of missing responses and other limitations of different impartation methods in relation to our analysis [32].

The low level of response rate to our study may be attributed to various reasons; previous studies have shown that students who are more vulnerable socioeconomically are less likely to participate in surveys [33]. It is possible that students who experienced stress due to the COVID-19 pandemic were also more likely to respond to the survey than students who did not experience stress. In addition, due to the online nature of the survey, responses may be biased toward students with good internet connectivity. Hence, sampling, response, and non-response bias may not be excluded. Furthermore, the generalizability of our findings may be limited due to potential differences between the international student population and the circumstances faced by students at Texas A&M University compared to those at other universities in the United States. Lastly, the pandemic and its impact are constantly evolving and dynamic; therefore, the validity of our findings may not be relevant in the long term. Nonetheless, lessons and insights gained now may be applied to forestall future similar pandemic or crisis scenarios.

## Conclusions

Our analysis showed that the COVID-19 pandemic posed a significant challenge to international students at TAMU across all three domains examined. The pandemic posed significant challenges to the students’ mental health as well as financial and academic well-being. Furthermore, respondents’ mental health challenges were further aggravated by academic and financial difficulties, such as the perception that the pandemic had negatively impacted their academic performance and their inability to pay their bills. Even though the majority of study participants indicated that they were satisfied with the University’s response to the pandemic, the government and the University need to provide more efficient and robust social and financial support to international students during crises like public health emergencies.
